# Analysis of Real-Time Face-Verification Methods for Surveillance Applications

**DOI:** 10.3390/jimaging9020021

**Published:** 2023-01-18

**Authors:** Filiberto Perez-Montes, Jesus Olivares-Mercado, Gabriel Sanchez-Perez, Gibran Benitez-Garcia, Lidia Prudente-Tixteco, Osvaldo Lopez-Garcia

**Affiliations:** 1 Instituto Politecnico Nacional, ESIME Culhuacan, Mexico City 04440, Mexico; 2 Graduate School of Informatics and Engineering, The University of Electro-Communications, Tokyo 182-8585, Japan

**Keywords:** face verification, lightweight face recognition, video surveillance, MobileFaceNet, EfficientNet, GhostNet

## Abstract

In the last decade, face-recognition and -verification methods based on deep learning have increasingly used deeper and more complex architectures to obtain state-of-the-art (SOTA) accuracy. Hence, these architectures are limited to powerful devices that can handle heavy computational resources. Conversely, lightweight and efficient methods have recently been proposed to achieve real-time performance on limited devices and embedded systems. However, real-time face-verification methods struggle with problems usually solved by their heavy counterparts—for example, illumination changes, occlusions, face rotation, and distance to the subject. These challenges are strongly related to surveillance applications that deal with low-resolution face images under unconstrained conditions. Therefore, this paper compares three SOTA real-time face-verification methods for coping with specific problems in surveillance applications. To this end, we created an evaluation subset from two available datasets consisting of 3000 face images presenting face rotation and low-resolution problems. We defined five groups of face rotation with five levels of resolutions that can appear in common surveillance scenarios. With our evaluation subset, we methodically evaluated the face-verification accuracy of MobileFaceNet, EfficientNet-B0, and GhostNet. Furthermore, we also evaluated them with conventional datasets, such as Cross-Pose LFW and QMUL-SurvFace. When examining the experimental results of the three mentioned datasets, we found that EfficientNet-B0 could deal with both surveillance problems, but MobileFaceNet was better at handling extreme face rotation over 80 degrees.

## 1. Introduction

Biometric recognition has played an important role in different application fields in recent decades. Frequent examples include face, iris, voice, palm, and fingerprint recognition [1]. One of the most widely used methods is facial recognition, which has experienced gains in its development in the last decade, with improvements in face processing, detection, and recognition [2]. Its primary objective is identifying which faces belong to individual identities within a dataset. On the other hand, face verification consists of analyzing the facial features of an image to determine if it belongs to the person it claims to be. Facial recognition and verification have shared problems related to illumination changes, occlusions, face rotation, and distance to the subject. These challenges are strongly related to video-surveillance applications; hence, the trending computer vision solution of deep learning can be used to address the mentioned problems. Deep neural networks (DNNs) are composed of several hidden layers with millions of artificial neurons connected and running parallel to handle a large amount of data [3]. Among DNNs, convolutional neural networks (CNNs) are the best-fitting option for image classification and object detection [3].

Currently, CNNs are more frequently used than traditional feature-extraction methods for face recognition, as they can solve common related issues such as changes in facial expressions, illumination, poses, low resolution, and occlusion [1]. CNNs are commonly built with complex architectures and high computational costs [4], with examples such as DeepFace [5], FaceNet [6], ArcFace [7], and MagFace [8]. Due to the huge amount of memory that these methods require, their applications are not designed to work in real-time on embedded devices with limited resources [4,9]. Therefore, lightweight CNN architectures have arisen that cover some of the mentioned requirements [9]. MobileFaceNet [10], EfficientNet-B0 [11], and GhostNet [12] are some of the lightweight architectures employed for face recognition and verification. Nonetheless, these methods struggle with problems usually solved by their heavy and more complex counterparts, such as face rotation and low-level face inputs.

The main contributions of this paper are three-fold: In this paper, we present an analysis of current SOTA methods for face verification based on lightweight architectures. The analysis specifically focuses on the problems of different facial rotations and low resolution present in video-surveillance camera applications. The SOTA methods used in the analysis include the aforementioned MobileFaceNet [10], EfficientNet-B0 [11], and GhostNet [12], as they are lightweight architectures that can be implemented in real-time and limited embedded devices. The datasets used to test methods were Cross-Pose LFW (CPLFW) [13] and QMUL-SurvFace [14], as they include facial images in different poses and low-resolution images, which are the problems analyzed in the present work. Furthermore, to methodically analyze the effect of face rotation and low-resolution problems, we propose an evaluation subset with 3000 facial images including the combination of the CPLFW [13] and Celebrities in Frontal-Profile in the Wild (CFPW) [15] datasets. We specifically define five groups of face rotation degrees with five levels of resolution that appear in common surveillance scenarios. With our complete analysis and based on the three datasets employed, we found that EfficientNet-B0 can deal with rotation and resolution problems, while MobileFaceNet is better at handling extreme face rotation over 80 degrees. The main contributions of this paper are three-fold:

- An evaluation subset with 3000 facial images obtained from CPLFW [13] and CFPW [15] was divided into five intervals of rotation degree and five resolution levels to evaluate rotation and resolution variations methodically.
- An analysis of three SOTA lightweight architectures (MobileFaceNet [10], EfficientNet-B0 [11], and GhostNet [12]) was carried out to deal with face-verification problems on conventional datasets (CPLFW [13] and QMUL-SurvFace [14]).
- A methodical analysis of the effect of facial rotation and low resolution was conducted for the face verification of the three aforementioned architectures.

## 2. Related Work

### 2.1. Datasets

This section presents conventional datasets divided into training and evaluation subsets conventionally used to test face-recognition and -verification methods. Training datasets usually include huge numbers of images containing information that the system should use in the learning process (variations in pose, light, occlusions, etc.). On the other hand, evaluation datasets focus on images with real-life conditions that attempt to emulate the ultimate face-recognition/-verification applications. In this paper, we introduced an evaluation subset that emulates security-video-surveillance applications, so one face is in the frontal view, and the other has a rotation angle. Using this methodology, we can evaluate face verification with five intervals of rotation angles and five resolution levels.

#### 2.1.1. Training Datasets

As mentioned, training datasets need large amounts of data with a robust distribution containing many identities. Here, we present some datasets commonly used to train CNN architectures. CASIA-WebFace (2014) [16] comprises 494,414 images from 10,575 identities with different facial rotations. VGG-Face (2015) [17] comprises 2622 identities with a total of 2.6 million images with different facial rotations and inclinations. MS-Celeb-1M (2016) [18] includes 100,000 identities with 100 pictures each, forming 10 million images with different poses and lighting. GANFaces-5M (2018) [19], with 10,000 identities, has five million images and is entirely made up of synthetic images with different expressions, poses, and lighting. One of the most-recent large-scale datasets is WebFace260M (2021) [20], which has four million identities and a total of 260 million images. It is worth noting that the pre-trained models used in this paper were trained with the MS-Celeb-1M-v1c [21] dataset, which consists of 3,923,399 aligned images (86,876 identities) cleaned from the standard MS-Celeb-1M [18] dataset.

#### 2.1.2. Evaluation Datasets

There are many public and private datasets available for face-verification assessment. However, this paper focuses on datasets that include significant sample variations related to face rotation and distance to the subject (low-resolution faces). Labeled Faces in the Wild (2007) [22], well-known as the LFW dataset, includes 5749 identities, in which 1680 have 2 or more images, with a total of 13,233 images showing different poses, expressions, and lighting. Multi-PIE (2010) [23] consists of 750,000 images from 337 identities with different poses, expressions, and lighting. Surveillance Cameras face (2011) [24] involves 130 identities with 4160 images with different facial resolutions resulting from varying the distance to the subject, and the images were collected from surveillance cameras. Trillion-Pairs [21] (2019) consists of 5700 identities with a total of 274,000 images with different ages and poses. All these datasets are frequently used in the literature to obtain facial-verification performance in general. However, we specifically focus on two problems related to surveillance applications: face rotation and distance to the subject. Therefore, the datasets chosen for our analysis were Celebrities in Frontal-Profile in the Wild (CFPW) [15], Cross-Pose LFW (CPLFW) [13], and QMUL-SurvFace [14], which will be detailed in the Experimental Results Section.

### 2.2. Face-Recognition Methods

Over the years, different CNN-based methods have been developed for face recognition and verification. Specifically, face verification in unconstrained environments is the primary task when evaluating the performance of conventional face-recognition systems [25,26]. While older approaches can obtain outstanding results in controlled environments, they tend to degrade significantly when real-life variations in facial pose, resolution, illumination, and occlusions are encountered [26,27]. To tackle these latent problems, Schroff et al. proposed a CNN-based approach called FaceNet [6], which is a conventional architecture that obtains compact 128D embedding based on a triplet loss function. FaceNet proved that the loss function is crucial in deep feature learning because it significantly improved face-recognition performance by penalizing the distance between negative and positive embeddings.

In the same way, several margin-based functions have been employed to regulate training and improve feature discrimination [28]. For instance, the A-SoftMax loss function with an angular margin was employed using the SphereFace [29] method. CosFace [30] uses the large margin cosine loss function to learn highly discriminative features. ArcFace [7] introduced an additive angular margin to maximize intraclass similarity and interclass diversity. AdaCos [31] proposes an adaptive scale parameter to automatically strengthen the training supervision using a cosine-based loss function. Similarly, MV-Softmax [32] adaptively emphasizes the misclassified feature vectors to guide the training, compiling feature-margin and feature-mining advantages in a single loss function. ElasticFace [33] relaxes the fixed penalty margin constraint to enable flexibility in class separability. In a more recent approach, MagFace [8] introduces an adaptive mechanism to learn a structured feature distribution within each class by pulling easy samples to class centers while pushing complex samples away.

On the other hand, there are works in the literature that propose CNN architectures or complete systems to solve specific face-recognition problems. For example, to tackle the pose-variation problem, Zhao et al. [34] used a generative adversarial network (GAN) to synthesize the frontal view of the face with pose variation. Ju et al. [35] proposed a Complete Face Recovery GAN (CFR-GAN) to restore collapsed textures, occlusion, and rotation. Likewise, to solve the problem of low-resolution faces, Nam et al. [36] introduced PSI-CNN, which uses a generic CNN architecture based on scale-invariant pyramids that can learn information at a different level in low-resolution images. Shahbakhsh and Hassanpou [37] presented a GAN to consider the image edges, which reconstructs the details to preserve the facial structure. Some works solve both the aforementioned problems of facial recognition in video-surveillance camera applications. For instance, Sayan et al. [38] used a multimodal recognition system that extracts the frontal view while walking and applies it to low-resolution facial images. Mishra et al. [39] introduced a multiscale parallel deep CNN to solve problems in low- and high-resolution images. Nadeem et al. [40] proposed integrating frontal and profile face image recognition using different CNNs in parallel, combining their predictions based on a single voting scheme.

The mentioned architectures and frameworks usually add extra parameters and computation to conventional CNNs, which limits their operability on limited devices and embedded systems. Therefore, in this paper, we focused our analysis on lightweight architectures that do not employ external blocks or add-ons. Specifically, we analyzed the performance of MobileFaceNet [10], EfficientNet-B0 [11], and GhostNet [12], which are trained with a cutting-edge loss function (MV-Softmax [32]), and their architectural details are described in the following section. It is worth noting that, to the best of our knowledge, there have been no previous analyses of real-time face-verification methods coupling the problems of security-video-surveillance applications, such as face rotation and low dimensionality.

## 3. Face Recognition in Real-Time

We considered the number of parameters and multiply–accumulate operations (MACs) to choose the real-time face-recognition methods for our analysis. Specifically, we limited our search to architectures that have about 30 M params. and about 200 M MACs. In this case, we ensured that they could be applied on limited devices and embedded systems. Thus, the three methods chosen are detailed below.

### 3.1. MobileFaceNet

**Figure 1:**
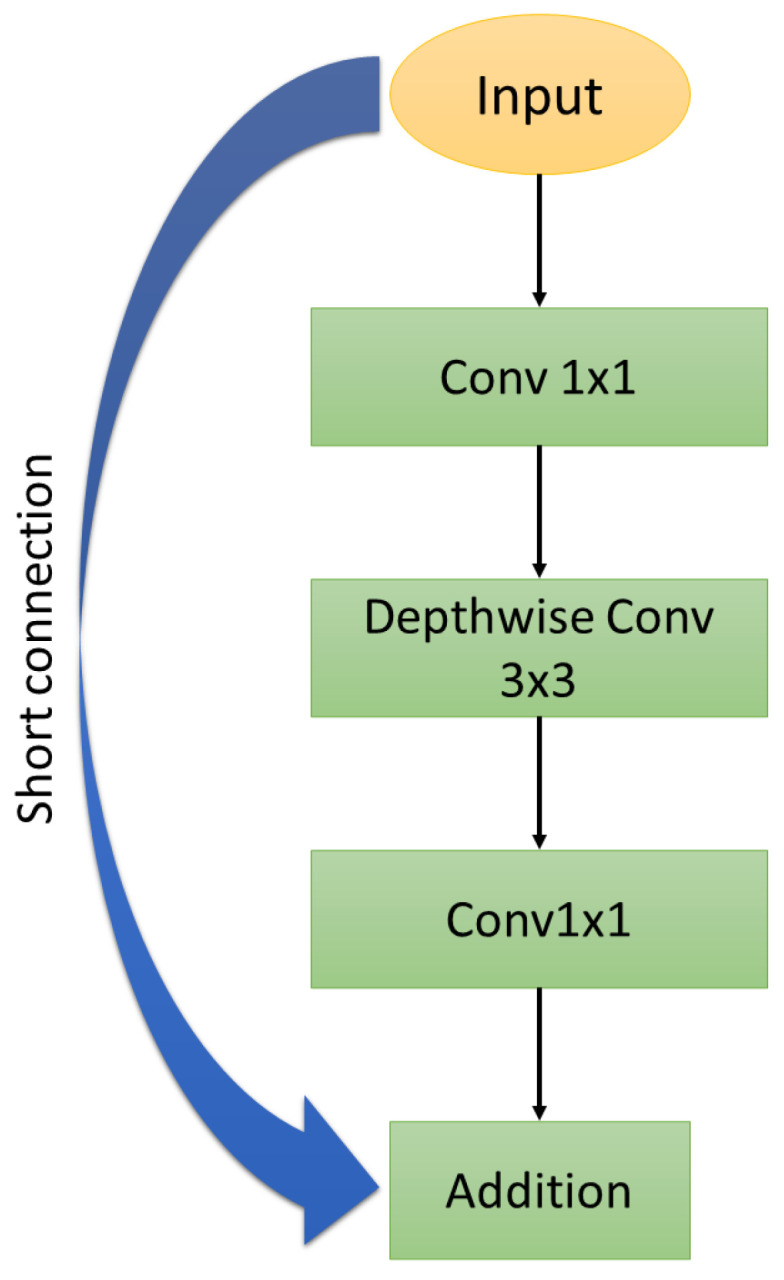
Bottleneck residual blocks in MobileFaceNet [41].

In 2018, Cheng et al. [10] proposed MobileFaceNet (1.2 M params. and 228 M MACs), which is based on the inverted residual bottlenecks introduced by MobileNetV2 [41], with small expansion factors as its main building blocks. The residual bottleneck block contains a three-layer convolution with direct access to the bottleneck connection, as shown in Figure 1. The depth-separable convolutions of MobileNetV1 [41] are used to reduce the size and complexity of the network [10]. In addition, the architecture uses the nonlinear activation function PReLU, helping face-verification performance. One of the main contributions of MobileFaceNet is the replacement of the global average pooling (GAPool) layer with the global depth convolutional layer (GDConv), which can obtain a more discriminating face representation. The GDConv layer deals with different levels of importance of different output feature maps, as it generates a 512-dimensional facial feature vector. GDConv is represented by:

(1) $$G_{m}=\;\sum_{i,j}K_{i,j,m}\;\cdot \;F_{i,j,m}$$

where *K* is a depth convolutional kernel of size W×H×M, *F* is the input feature map of size W×H×M, and (i,j) is the spatial dimension in *K* and *F*. *M* refers to the channel index, and $G_{m}$ is the *m*-th channel in *G*. *G* is the output of size 1×1×M. *W* is the spatial width. *H* is the spacial height of a feature map. *M* is the number of input channels. The GDConv layer has a computational cost assigned by

(2) W·H·M.

The MobileFaceNet architecture is shown in Table 1. The expansion multiplier is defined as ***t***. ***c*** is the number of channels. ***n*** is the blocked repeated time. ***s*** is the step stride [10]. It is worth noting that MobileFaceNet has been tested and employed in different face-recognition applications, such as in [42,43,44].

### 3.2. EfficientNet

**Figure 2:**
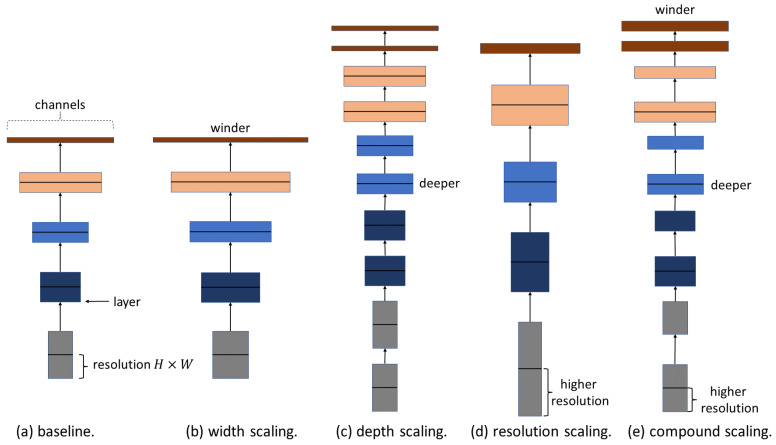
Architectural representation of standard and composite scaling methods. (**a**) ConvNet generic, (**b**) ConvNet with width scaling, (**c**) ConvNet with Depth scaling, (**d**) ConvNet with resolution scaling, and (**e**) ConvNet with compound scaling.

In 2019, Tan and Le [11] introduced EfficientNet (33 M params. and 78 M MACs), which combines a neural architecture search (NAS) with a composite scaling method to optimize the training speed and efficiency jointly. The idea of EffcientNet is to expand the width, depth, and resolution of the grid through the composite-scaling method, as shown in Figure 2e. In addition, a single variable is used to uniformly scale the width, depth, and resolution of the network [11]. The following equations show the composite scaling method:

(3) $$\begin{array}{c} Depth:d=\alpha^{\phi }\\ Width:w=\beta^{\phi }\\ Resolution:r=\gamma^{\phi }\\ s.t.\;\alpha \;\circ \beta_{2}\circ \gamma_{2}\approx 2\\ \alpha \geq 1,\;\beta \geq 1,\;\gamma \geq 1 \end{array}$$

where α, β, and γ are the distribution coefficients of the network depth, width, and resolution, respectively (all found by the NAS using MBConv blocks). A composite coefficient phi is used to find the alpha, beta, and gamma parameters that maximize the recognition accuracy. It is important to note that phi is adjusted according to the desirable computational resources [11].

The reference network of EfficientNet-B0 is obtained by calculating the coefficients α, β, and γ using a small grid search when ϕ=1. More complex versions of EfficientNet have been proposed by scaling the reference network with different ϕ (EfficientNet-B1-7) [11].

The EfficientNet-B0 architecture is shown in Table 2. The number of output feature channels and convolutional layers of each stage are shown as channels and layers, respectively. EfficientNet mainly comprises mobile inverted bottleneck convolution (MBConv1, MBConv6), standard convolutional layers, pooling layers, and one fully connected layer [11].

### 3.3. GhostNet

**Figure 3:**
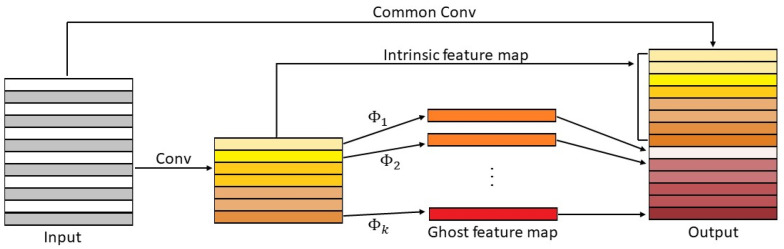
Representation of a GhostNet module [12].

In 2020, Han et al. [12] presented GhostNet (27 M params. and 194 M MACs), mainly constituted by the proposed Ghost modules. The main contribution of these modules is to substitute a significant part of the convolutional filters with a series of linear transformations. Ghost feature maps are generated by economic operations, saving computation from the standard convolutions. A Ghost module is shown in Figure 3, and it can be expressed by

(4) $$Y^{\prime }=\;X\;\times F^{\prime }$$

where $Y^{\prime }$ is the *m* intrinsic feature map generated by the primary convolution, *X* is the input feature map, × is the convolution operation, and $F^{\prime }$ is the kernel size of the convolutional filter. Thus, the feature maps are given by

(5) $$y_{ij}\;=\;\Phi_{i,j}\left({y^{\prime }}_{i}\right),\;\;\;\forall \;i\;=\;1,\;\cdots ,m,\;\;\;j\;=\;1,\;\cdots ,\;s,$$

$\Phi_{i,j}$ is the *j*-th linear operation used to generate the *j*-th Ghost feature map. $y_{ij}$, except for the last $\Phi_{i,s}$, is the identity mapping used to preserve the intrinsic feature maps. ${y^{\prime }}_{i}$ is the *i*-th intrinsic feature map in $Y^{\prime }$. The Ghost module is plug-and-play and can be used to update existing CNNs [12].

The GhostNet architecture is shown in Table 3, where ***t*** denotes the expansion size, ***c*** is the number of output channels, SE indicates whether the squeeze-and-excitation (SE) module is used, and ***stride*** is the number of steps that the neural network filter moves in the image [12]. Bottlenecks are gathered according to the sizes of the input feature maps [12]. The average pooling and a convolutional layer are used to transform the feature maps into a 1280-dimensional vector for the classification [12].

## 4. Experiment Setup

This section presents the implementation details used for evaluating the MobileFaceNet, EfficientNet-B0, and GhostNet architectures. We specifically compared their performance in face verification, where the conventional CPLFW and QMUL-SurvFace datasets were first used to analyze scenarios where face rotation and low-resolution images appeared in video surveillance cameras (Experiment 1). In addition, the proposed evaluation subset was used to methodologically analyze the impact of face rotation using a particular rotation degree group and low resolution by using specific image sizes. The main goal of our analysis was to understand how images with rotation or low resolution affect the facial-verification performance of the SOTA lightweight architectures (Experiment 2).

### 4.1. Implementation Details

All experiments were run on a computer with a 7th-generation Intel Core i7 processor, 32 GB of RAM, and a single NVIDIA GTX 1060 GPU. We used Python 3.10, Torch 1.12.0, and Torchvision 0.13.0 with CUDA 11.3. To obtain the verification accuracy, we employed the pre-trained models (MobileFaceNet [10], EfficientNet-B0 [11], and GhostNet [12]) shared by the FaceX-Zoo repository [45]. These models were trained with the MS-Celeb1M-v1c [21] dataset with a stochastic gradient descent (SGD) optimizer, a momentum of 0.9, and the MV-Softmax [32] loss function. The training batch size was 512, with a total of 18 epochs and a learning rate initialized at 0.1 and divided by 10 at Epochs 10, 13, and 16. To perform the test with the CPLFW dataset, QMUL-SurvFace, and the proposed evaluation subset, the images were normalized to 112 × 112 pixels using the same parameters from [45].

### 4.2. Datasets

The CPLFW [13] dataset contains 11,652 images of 3930 identities at a resolution of 250 × 250 pixels with different facial pose variations. We used 6000 total pairs (3000 positive and 3000 negative pairs) for the evaluation. The QMUL-SurvFace [14] dataset comprises 463,507 video-surveillance images with 15,573 identities. Out of 10,638 identities, 2 or more images were included with resolutions between 6 × 5 and 124 × 106 pixels. The average resolution was 24 × 20 pixels and can be used for facial verification and identification [14]. A total of 10,640 pairs (5320 positive and 5320 negative) were used in our evaluation.

To methodologically analyze face-verification performance in scenarios where variations such as face rotation and low resolutions are present, we designed an evaluation subset using the CPLFW [13] and CFPW [15] datasets. The CFPW [15] dataset has 7000 images from 500 identities, with 10 frontal and 4 profile pictures each. For the construction of our evaluation subset, we used a facial-pose-estimation method (6DRepNet [46]) to determine the rotation degree and thus divide the images into 5 angle intervals ([0°; 20°], [20°; 40°], [40°; 60°], [60°; 80°], and [80°; 180°]).

The facial-pose estimation method 6DRepNet [46] is based on a CNN and uses a 6D continuous rotation matrix for compressed regression. Thus, it can learn the entire facial rotation appearance using a geodesic loss to penalize the network with respect to the special orthogonal group SO(3) geometry. The publicly available code of 6DRepNet [46] was used to obtain the rotation angle from all faces.

**Figure 4:**
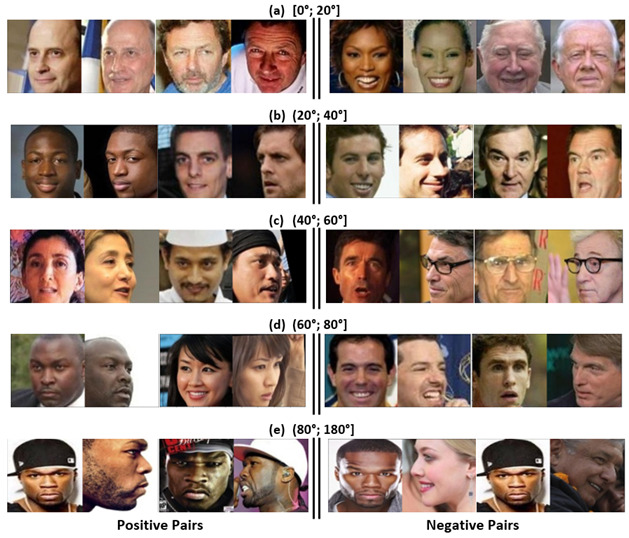
Examples of the pairs chosen for the proposed evaluation subset.

**Figure 5:**
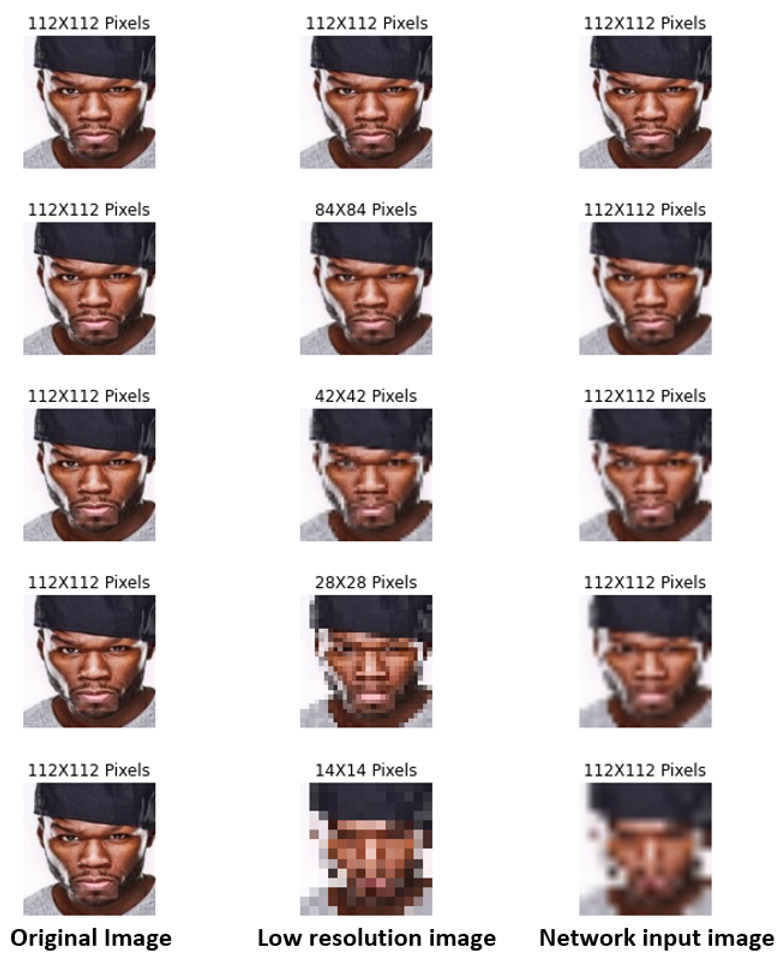
Example of the five levels of resolution in the proposed evaluation subset.

It is worth noting that, from each pair of images in our evaluation subset, we specifically selected one image in frontal view and another with a rotation angle. In this way, we emulated security-video-surveillance applications. Table 4 shows the numbers of our evaluation subset, with 200 pairs for the intervals of [0°; 20°], [20°; 40°], [40°; 60°], and [60°; 80°] and 700 pairs of [80°; 180°]. Figure 4 shows some examples of the pairs included. Furthermore, to overcome the challenges of distance to the subject in the video-surveillance cameras, we resized the resolution of our evaluation subset. Figure 5 shows an example of the five resolution levels, their equivalent at the standard resolution, and the resized input to the three methods.

## 5. Experimental Results

### 5.1. Evaluation with Conventional Datasets

In the first experiment, we analyzed the performance of lightweight architectures with 6000 pairs from CPLFW [13]. Table 5 shows the facial-verification performance for the three pre-trained models.

**Figure 6:**
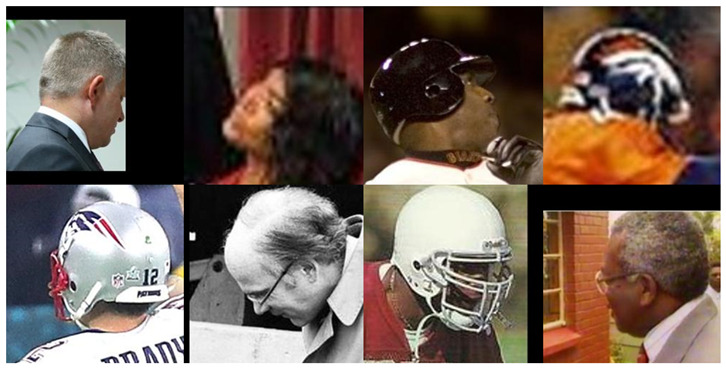
Examples of the occlusions found in the faces not included.

Table 5 shows that, for the CPLFW [13] dataset, the EffcientNet-B0 [11] model has the best verification performance compared to the other two models. To analyze the facial-verification performance using angle rotation, we also used the 6DRepNet [46] method. Unfortunately, we could only obtain 5864 pairs. The pairs not included were misdetections caused by heavy occlusions generated by rotations greater than 90°, soccer helmets, cropped images, etc. Figure 6 shows examples of the occlusions found in the faces not included. We grouped the detected pairs by the angle difference between each image pair. Hence, we also defined five intervals, [0°; 20°], [20°; 40°], [40°; 60°], [60°; 80°], and [80°; 180°]. Table 6 shows the results of the verification performance for each angle interval.

**Figure 7:**
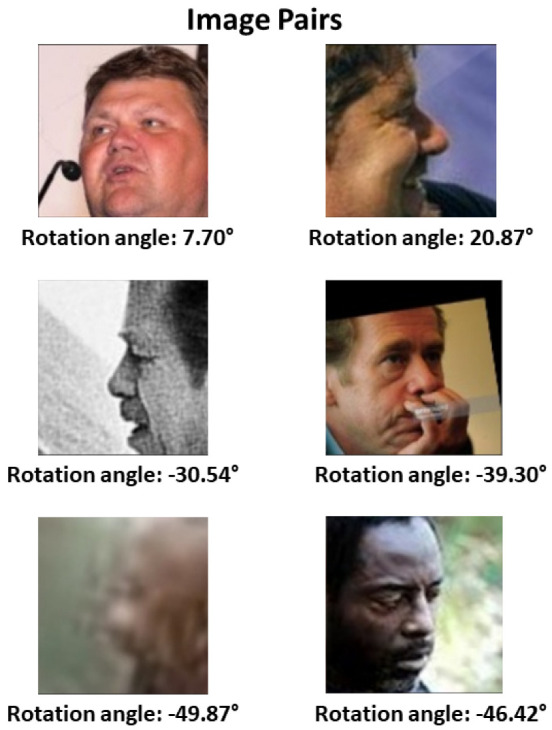
Example of image pairs with facial pose estimation error (attributed to the [0°; 20°] interval).

As we can see in Table 6, EfficientNet-B0 [11] has the best verification performance for all intervals. It is important to note that the accuracy of the [0°; 20°] interval is lower than that of [20°; 40°]. This inconsistency in the results can be attributed to angle-detection problems. Figure 7 shows examples of image pairs that are supposed to belong to the [0°; 20°] interval, where we can see the apparent misdetection problems. However, with this test, we can see that, in general, if the rotation angle increases, the verification accuracy decreases.

**Figure 8:**
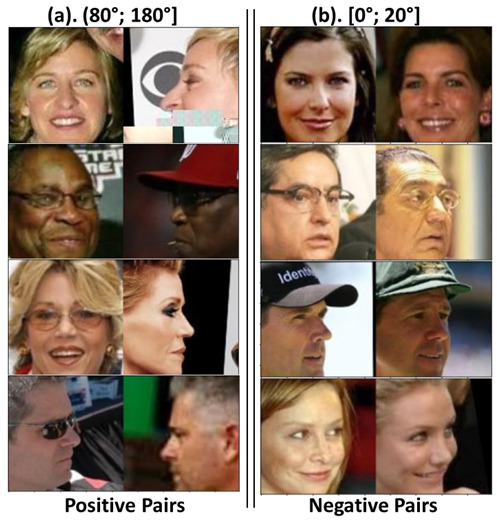
Example of CPLFW image pairs incorrectly classified by EfficientNet-B0.

Figure 8 shows examples of image pairs incorrectly classified by EfficientNet-B0. In these two intervals, the images present occlusions (images with missing pixels in the face, glasses, and cap) and extreme rotations, making facial verification difficult.

**Figure 9:**
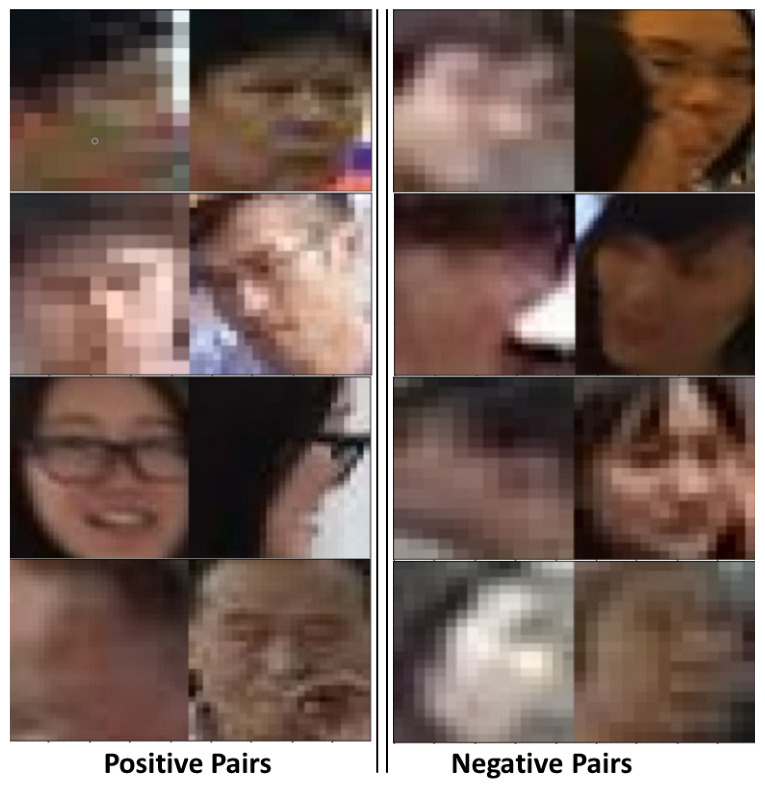
Example of QMUL-SurvFace image pairs incorrectly classified by EfficientNet-B0.

Next, we also analyzed the performance of the THREE methods using 10,640 image pairs from the challenging QMUL-SurvFace dataset [14]. Table 7 shows the verification performance, where EffcientNet-B0 achieved the best results again. It is important to note that the results of all methods are low due to the image quality, capture distance, occlusions, and extreme rotations. Figure 9 shows examples of image pairs incorrectly classified by EfficientNet-B0.

### 5.2. Evaluation with the Proposed Evaluation Subset

We started this test by analyzing the performance of the three methods with 1500 pairs from the proposed evaluation subset. Table 8 shows the face-verification performance, where MobileFaceNet [10] surprisingly had the best verification performance. We also analyzed the performance of all methods with facial rotations divided into five angle intervals. Table 9 shows the verification performance of each interval.

**Figure 10:**
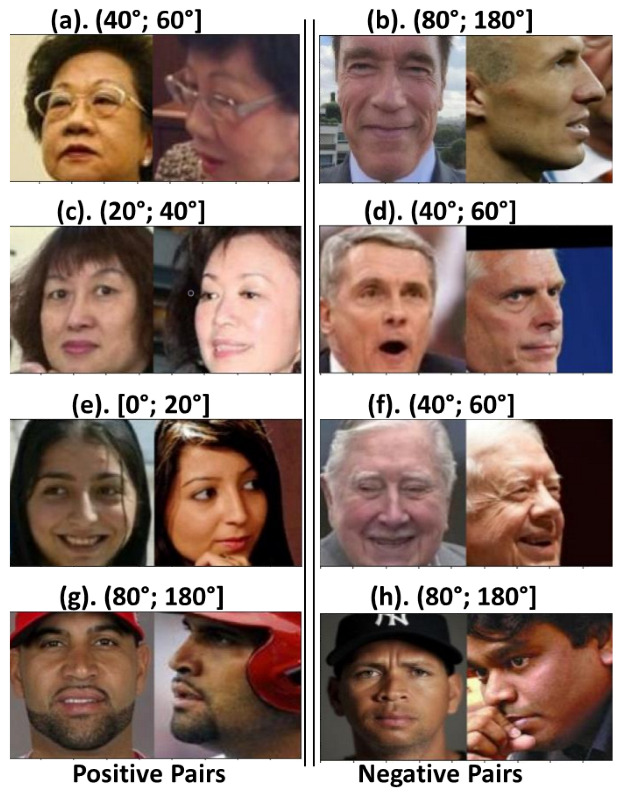
Example of evaluation subset pairs incorrectly classified by MobileFaceNet.

We can see from Table 9 that MobileFaceNet [10] has the best verification performance in the intervals [0°; 20°], [20°; 40°], and [80°; 180°]. Meanwhile, EfficientNet-B0 [11] has the best verification performance for [20°; 40°], [40°; 60°], and [60°; 80°]. Thus, MobileFaceNet [10] has the best general accuracy, and it is the best method for handling extreme facial rotation for angles greater than 80°. It was found that the verification accuracy decreased as the rotation angle increased in each interval because all of the images were at an extreme angle, and the feature vector had less information to provide. Figure 10 shows examples of image pairs misclassified by MobileFaceNet.

Furthermore, we analyzed the performance of the three methods with the resolutions of 14^2^, 28^2^, 42^2^, 84^2^, and 112^2^ pixels in our evaluation subset. Table 10 shows the obtained results of the verification accuracy with different resolution levels. MobileFaceNet [10] achieved the best results for 28 × 28 to 112 × 112 pixels. This may be attributed to the richness of the feature vector generated with the GDConv of the architecture. On the other hand, EfficientNet-B0 [11] had the best verification performance for 14 × 14 pixels, which can be attributed to the specific filter sizes found by the NAS procedure.

**Figure 11:**
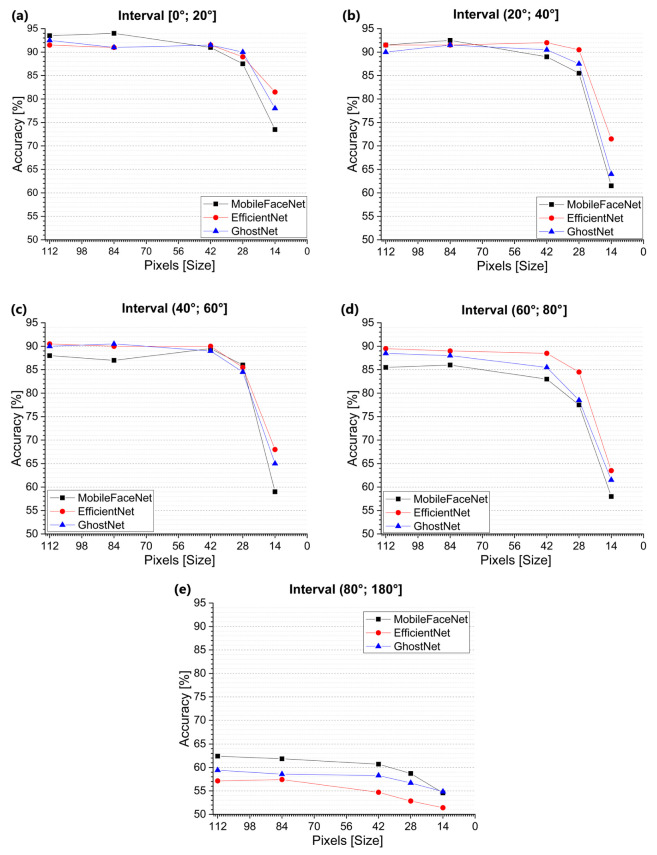
Plot results for each angle range with different resolution levels, where the following intervals are shown: (**a**) $\left[0^{\circ };20^{\circ }\right]$, (**b**) $\left[20^{\circ };40^{\circ }\right]$, (**c**) $\left[40^{\circ };60^{\circ }\right]$, (**d**) $\left[60^{\circ };80^{\circ }\right]$, and (**e**) $\left[80^{\circ };180^{\circ }\right]$. Pixel resolutions of 14 × 14, 28 × 28, 42 × 42, 84 × 84, and 112 × 112 are used in all intervals.

We also analyzed the facial rotation together with different resolutions. Figure 11 shows plots for each angle interval with different resolution levels. In Figure 11a, it can be seen that MobileFaceNet [10] had the best performance when working with images equal to or greater than 84 pixels, EffcientNet-B0 [11] was the best for images of 14 and 42 pixels, and GhostNet [12] was the best for images of 28 to 42 pixels. In Figure 11b, it can be seen that MobileFaceNet [10] had the best performance for working with images equal to or larger than 84 pixels, while EffcientNet-B0 [11] was the best for images from 14 to 42 and 112 pixels. Figure 11c shows that MobileFaceNet [10] had the best performance for images with 28 pixels; EffcientNet-B0 [11] was the best for 14, 42, and 112 pixels, and GhostNet [12] was the best for 84 pixels. Figure 11d shows that EffcientNet-B0 [11] achieved the best results for 14- to 112-pixel images. Figure 11e indicates that MobileFaceNet [10] had the best performance when working with 28- to 112-pixel images, while GhostNet [12] was the best for 14-pixel images.

**Figure 12:**
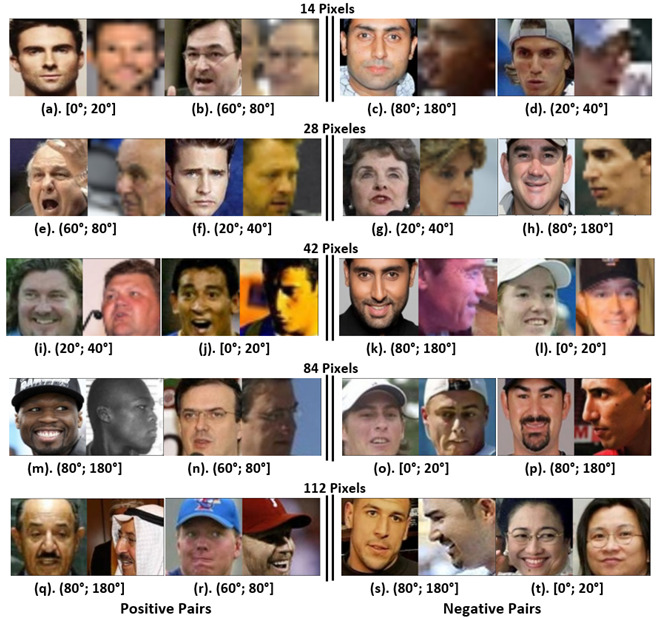
Examples of evaluation subset pairs per resolution interval incorrectly classified by MobileFaceNet.

In summary, EfficientNet-B0 [11] is the best method for working with 14 × 14-pixel images in all of the different intervals, except for the [80°;180°] interval. MobileFaceNet [10] with image resolutions from 28 × 28 to 112 × 112 pixels proved to be the best method to work in the interval [80°;180°], where extreme rotations are found. Figure 12 shows examples of image pairs misclassified by MobileFaceNet, where we can qualitatively corroborate the challenges for each angle and resolution interval.

Finally, in Table 11, we present the inference time of each method running on a single GPU (NVIDIA GTX 1060) and CPU (Intel Core i7). The time was averaged over 500 single passes of 112 × 112-pixel images. In this table, we can see that MobileFaceNet is the only approach that can surpass the real-time barrier of 30 FPS. However, all methods can run over 15 FPS, which is considered efficient on a CPU and low-cost GPU such as the GTX 1060.

## 6. Discussion

Based on the analysis using two conventional datasets, EfficientNet-B0 demonstrated that it could handle different facial rotations, prominent occlusions, illuminations, and low resolutions. This is because the mobile inverted bottleneck convolution in the first layer expands the channels and compresses them. Consequently, the layers with fewer channels skip connections to obtain discriminative feature maps to generalize facial features. Therefore, such features (facial contour, nose, eyes, eyebrows, mouth, etc.) can be enriched between each pair of images in training.

An evaluation subset was designed to analyze only the variations with different rotations and low resolutions to understand how the methods work with images that can be obtained in video-surveillance cameras. This evaluation subset has well-defined image pairs for each angle range and five resolution levels. EfficientNet-B0 proved to be the best method to work with resolutions of 14 × 14 pixels and a rotation of less than 80°. On the other hand, MobileFaceNet proved to be the best with extreme rotations (greater than 80°) with resolutions from 28 × 28 to 112 × 112 pixels. This might relate to the global depthwise convolutional modules responsible for obtaining rich feature maps in specific regions of the face. GhostNet, on average, did not perform well because Ghost modules lack features that better represent the face, which is attributed to the “cheap” features calculated by linear transformations instead of standard convolutional operations.

## 7. Conclusions

In this paper, we analyzed the real-time face-verification methods of MobileFaceNet, EfficientNet-B0, and GhostNet using datasets explicitly focusing on problems present in video-surveillance applications. We tested their performance on conventional datasets (CPLFW and QMUL-SurvFace) that also have different illuminations, occlusions, and facial expressions. In addition, we proposed an evaluation subset that focused only on the problems of facial rotation and low resolutions, divided into five angle intervals and five levels of resolution. The experimental results showed that, for resolutions of 14 × 14 pixels with angles less than 80°, EfficientNet-B0 was the best method. MobileFaceNet, at angles greater than 80° and with resolutions of 28 × 28 up to 112 × 112 pixels, proved to be the best method compared to the other two. Therefore, we can conclude that using the three mentioned datasets, EfficientNet-B0 can cope with facial rotation variations and low resolutions in general, while MobileFaceNet can cope with extreme rotations. Nonetheless, all analyzed methods can run on limited devices and embedded systems in real-time.

## Figures and Tables

**Table 1:** MobileFaceNet architecture [10].

Input	Operator	*t*	*c*	*n*	*s*
112^2^ × 3	Conv3×3	-	64	1	2
56^2^ × 64	Depthwise Conv3×3	-	64	1	1
56^2^ × 64	Bottleneck	2	64	5	2
28^2^ × 64	Bottleneck	4	128	1	2
14^2^ × 128	Bottleneck	2	128	6	1
14^2^ × 128	Bottleneck	4	128	1	2
7^2^ × 128	Bottleneck	2	128	2	1
7^2^ × 128	Conv1×1	-	512	1	1
7^2^ × 512	Linear GDConv7×7	-	512	1	1
1^2^ × 512	Linear Conv1×1	-	128	1	1

**Table 2:** EfficientNet-B0 architecture [11].

Stage	Operator	Resolution	Stride	# of Channels	Layers
1	Conv3×3	224 × 224	2	32	1
2	MBConv1, k3×3	112 × 112	1	16	1
3	MBConv6, k3×3	112 × 112	2	24	2
4	MBConv6, k5×5	56 × 56	2	40	2
5	MBConv6, k3×3	28 × 28	2	80	3
6	MBConv6, k5×5	14 × 14	1	112	3
7	MBConv6, k5×5	14 × 14	2	192	4
8	MBConv6, k3×3	7 × 7	1	320	1
9	Conv1×1 and Pooling and FC	7 × 7	1	1280	1

**Table 3:** GhostNet architecture [12].

Input	Operator	*t*	*c*	SE	*Stride*
224^2^ × 3	Conv2d3×3	-	16	-	2
112^2^ × 16	G-bneck	16	16	-	1
112^2^ × 16	G-bneck	48	24	-	2
56^2^ × 24	G-bneck	72	24	-	1
56^2^ × 24	G-bneck	72	40	1	2
28^2^ × 40	G-bneck	120	40	1	1
28^2^ × 40	G-bneck	240	180	-	2
14^2^ × 80	G-bneck	200	80	-	1
14^2^ × 80	G-bneck	184	80	-	1
14^2^ × 80	G-bneck	184	80	-	1
14^2^ × 80	G-bneck	480	112	1	1
14^2^ × 112	G-bneck	672	112	1	1
14^2^ × 112	G-bneck	672	160	1	2
7^2^ × 160	G-bneck	960	160	-	1
7^2^ × 160	G-bneck	960	160	1	1
7^2^ × 160	G-bneck	960	160	-	1
7^2^ × 160	G-bneck	960	160	1	1
7^2^ × 160	Conv2d1×1	-	960	-	1
7^2^ × 960	AvgPool7×7	-	-	-	-
1^2^ × 960	Conv2d1×1	-	1280	-	1

**Table 4:** Number of images and pairs per interval of each dataset used to build the proposed evaluation subset.

Intervals	# of CPLFW Images	# of CFPW Images	Total # of Images	# of Positive Pairs	# of Negative Pairs	Total # of Pairs
$\left[0^{\circ };20^{\circ }\right]$	326	74	400	100	100	200
$\left[20^{\circ };40^{\circ }\right]$	400	-	400	100	100	200
$\left[40^{\circ };60^{\circ }\right]$	400	-	400	100	100	200
$\left[60^{\circ };80^{\circ }\right]$	400	-	400	100	100	200
$\left[80^{\circ };180^{\circ }\right]$	-	1400	1400	350	350	700
**Total**	1526	1474	3000	750	750	1500

**Table 5:** Verification performance with the CPLFW [13] dataset.

Model	Accuracy (%)
MobileFaceNet	83.08
**EfficientNet-B0**	**85.16**
GhostNet	83.51

**Table 6:** Verification performance over the five intervals using the CPLFW [13] dataset.

Intervals	# of Pairs	MobileFaceNet (%)	EfficientNet-B0 (%)	GhostNet (%)
$\left[0^{\circ };20^{\circ }\right]$	1237	86.41	**87.55**	86.82
$\left[20^{\circ };40^{\circ }\right]$	1127	88.55	**89.17**	87.57
$\left[40^{\circ };60^{\circ }\right]$	1258	84.18	**87.28**	85.77
$\left[60^{\circ };80^{\circ }\right]$	1017	80.13	**83.77**	82.10
$\left[80^{\circ };180^{\circ }\right]$	1225	78.93	**81.06**	78.53

**Table 7:** Verification performance with the QMUL-SurvFace [14] dataset.

Model	Accuracy (%)
MobileFaceNet	63.78
**EfficientNet-B0**	**63.82**
GhostNet	62.58

**Table 8:** Verification performance with the proposed evaluation subset.

Model	Accuracy (%)
**MobileFaceNet**	**76.93**
EfficientNet-B0	75.06
GhostNet	75.86

**Table 9:** Verification performance over the five intervals with the proposed evaluation subset.

Intervals	# of Pairs	MobileFaceNet (%)	EfficientNet-B0 (%)	GhostNet (%)
$\left[0^{\circ };20^{\circ }\right]$	200	**93.50**	91.50	82.50
$\left[20^{\circ };40^{\circ }\right]$	200	**91.50**	**91.50**	90.00
$\left[40^{\circ };60^{\circ }\right]$	200	88.00	**90.50**	90.00
$\left[60^{\circ };80^{\circ }\right]$	200	85.00	**89.50**	88.50
$\left[80^{\circ };180^{\circ }\right]$	700	**62.42**	57.14	59.42

**Table 10:** Verification performance of the five resolution levels with the proposed evaluation subset.

Model	14-Pixel Accuracy (%)	28-Pixel Accuracy (%)	42-Pixel Accuracy (%)	84-Pixel Accuracy (%)	112-Pixel Accuracy (%)
MobileFaceNet	59.06	**72.26**	**75.33**	**76.80**	**76.93**
EfficientNet-B0	**61.93**	71.26	73.80	75.00	75.06
GhostNet	61.40	71.86	74.73	75.46	75.86

**Table 11:** Inference time.

Method	GPU (GTX 1060)	CPU (Intel Core i7)
MobileFaceNet	**27.0 ms (37.0 FPS)**	**29.9 ms (33.4 FPS)**
EfficientNet-B0	52.1 ms (19.2 FPS)	55.6 ms (18.0 FPS)
GhostNet	30.3 ms (32.9 FPS)	33.5 ms (29.9 FPS)

## Data Availability

Not applicable.
